# Establishment of a miRNA profile in paediatric HIV-1 patients and its potential as a biomarker for effectiveness of the combined antiretroviral therapy

**DOI:** 10.1038/s41598-021-03020-5

**Published:** 2021-12-06

**Authors:** Irene Consuegra, Samanta Gasco, María Jesús Serramía, José Luis Jiménez, Maria Jose Mellado, María Ángeles Muñoz-Fernández

**Affiliations:** 1 Instituto de Investigación Sanitaria Gregorio Marañón (IiSGM), Madrid, Spain; 2 Spanish HIV HGM BioBank, Madrid, Spain; 3 Laboratorio InmunoBiología Molecular, Head Immunology Section, (Hospital General Universitario Gregorio Marañón), C/Dr. Esquerdo 46, 28007 Madrid, Spain; 4 Plataforma-Laboratorio (IiSGM), Madrid, Spain; 5 General Pediatrics, Infectious and Tropical Diseases Department Hospital, Universitario La Paz, Madrid, Spain; 6 IdiPAZ, Madrid, Spain; 7 Translational Research Network in Pediatric Infectious Diseases (RITIP), Madrid, Spain; 8 Networking Research Center on Bioengineering, Biomaterials and Nanomedicine (CIBER-BBN), Madrid, Spain

**Keywords:** Paediatric research, Predictive markers

## Abstract

miRNAs have been extensively studied in pathological conditions, including viral infections, such as those provoked by HIV-1. Several cellular and circulating miRNAs are altered during HIV-1 infection, with either beneficial effects on host defenses or enhanced virus infectivity. Blood samples were collected in sterile EDTA tubes and plasma was separated and stored, as were PBMCs. RNA was isolated and reverse-transcribed. Finally, the miRNA gene expression profile was assessed using TaqMan Array Human microRNA Card A v2.0. A comprehensive statistical analysis was performed on the results obtained. This is the first study on miRNAs in HIV-1 paediatric patients, and a miRNA profile differentiating patients starting combination antiretroviral therapy (cART) at different times after HIV-1 diagnosis was established. Thirty-four miRNAs were observed to have different expression levels between the control group and the cART group. The data indicates the need to start cART as soon as possible after the establishment of HIV-1 infection to assure the best outcome possible. Finally, the selected 34 miRNAs may be used as biomarkers for prognosis and assessing therapy effectiveness. However, more research must be conducted to establish adequate quantitative correlations.

## Introduction

miRNAs are small noncoding RNAs (sncRNAs) encoded by eukaryotic genes present in intergenic regions or introns within the genome^1–3^. These small molecules form part of an intricate system regulating several cellular processes, including cell differentiation and proliferation, cell death, metabolic processes, and pathological conditions, such as cancer. The main function of miRNAs involves suppressing the expression of their target genes at the posttranslational level by binding to the 3’ UTR of their target mRNAs and either blocking translation or causing direct degradation^3^. Moreover, some miRNAs have also been found to enhance or regulate gene expression in a different manner in some specific situations and to participate in nuclear functions^4–7^.

miRNAs have been extensively studied in cancer. However, they can also shed light on processes related to infectious diseases, especially those affecting viral infections provoked by DNA and RNA viruses, such as human immunodeficiency virus type 1 (HIV-1)^8,9^. The organism is able to upregulate or downregulate specific miRNAs, affecting the machinery used by the invading viruses, either in a beneficial or detrimental way. These miRNAs can also directly target viral components^10,11^. Viruses can also modulate cellular miRNAs to their advantage and can potentially induce the biogenesis of their own miRNAs, called v-miRNAs, which are encoded by the viral genome or to inhibit cellular molecules targeting their own transcripts. Regardless, the actual functionality of v-miRNAs is still under debate^9,10^.

Several cellular miRNAs and circulating miRNAs have been found to be altered during the course of HIV-1 infection^8,12,13^. Some of these alterations are a response of the organism to attempt to control virus replication and propagation by indirectly modulating cellular proteins^12,14^. For example, immune cells can decrease expression of CCR5 and CXCR4 receptors, thus diminishing the ability of HIV-1 to infect target cells^13^. Conversely, the expression levels of other host miRNAs can be altered by the virus to use them to its advantage, usually by sequestering or inhibiting miRNAs regulating the cellular machinery^11,12^. Moreover, computational analysis has unveiled the potential existence of some host miRNAs that may directly affect and downregulate HIV-1 transcripts by binding to the virus itself, though the real extent of their functionality remains to be confirmed in vivo^8,12^. These direct-effect miRNAs are thought to function by binding to HIV-1 transcripts coding for viral proteins, such as envelope (env), polymerase (pol), viral infectivity factor (vif), group-specific antigen (gag) and tat^13^. For example, miR-28, miR-125b, miR-150, miR-223 and miR-382 were observed to target the 3´ ends of HIV-1 mRNA, and their expression levels were especially high in resting CD4 + T cells. This might explain the resistance of these cells to HIV-1 infection in comparison to activated CD4 + T-cells, which show downregulation of these five miRNAs. Consequently, these miRNAs have important implications in the regulation of HIV-1 latency^15^. Although there is controversy about v-miRNAs that may be encoded by the HIV-1 genome, it has been shown that HIV-1 proteins are able to induce some v-miRNAs that can alter host miRNA expression levels or affect several cellular functions^16^. For example, the tat gene induces miR-TAR, a miRNA that targets phosphatase and tensing homologue (PTEN), thus leading to indirect upregulation of miR-21 and miR-22. This alteration of their expression levels renders infected CD4 + T cells more resistant to apoptosis, enhancing persistence and expansion of HIV-1^17^. The nef gene has also been found to contain a potential sequence for a v-miRNA that would enhance HIV-1 infectivity^18^. In view of all the research on miRNAs related to the virus-host relationship, it seems clear that these sncRNAs offer an interesting study target in terms of HIV-infection research, as they may be used as biomarkers and therapeutic targets.

HIV-1 infection in newborns, infants and children have unique characteristics, especially since the immune system is still immature and develops in this age range. The way that HIV-1 acts and the immune system responds to its presence is quite different from that observed for patients first infected in their adult life^19,20^. The innate immune system is most likely to act against the HIV-1 infection in paediatric patients, given its broad and non-specific nature and the immature state of the adaptive immune system, which would have little to no previous encounters with antigens to mount an effective defense response against exogenous pathogens^19,21^. Moreover, the human leukocyte antigen (HLA) alleles inherited from the mother may influence HIV infection establishment and progression in HIV-1 paediatric patients, as the virus transmitted from mother to child might have “learned” how to attack cells with those HLAs^21^. The importance of establishing a miRNA profile and monitoring changes in it has been previously described for adult HIV-1-infected patients with different viral loads and progression rates^22–25^. miRNA expression levels can also offer relevant data in the case of HIV-1 paediatric patients, as they have their own characteristics. Thus, it is crucial to have useful tools to predict prognosis and effectiveness of the combination therapy applied to this especially vulnerable paediatric population.

The aim of this work was to establish the first miRNA profile study in peripheral blood mononuclear cells (PBMCs) of HIV-1-infected paediatric patients treated with combined antiretroviral therapy (cART) and undetectable viremia in blood and to distinguish miRNA expression levels based on the time of cART initiation. This study has been previously performed in adult HIV-1 patients but not in paediatric HIV-1 patients^26^. Consequently, the results obtained are valuable because they identify a specific miRNA signature that discriminates between cART-treated and untreated paediatric patients and assesses treatment effectiveness against HIV-1 infection.

## Results

### Study desing

Fourteen samples from paediatric HIV-1-infected patients with undetectable viral loads in blood and three samples from control individuals were obtained. The children’s ages ranged from 3 to 15 years old at the time of collection, and they acquired HIV-1 infection by vertical transmission from their mothers. Four groups were established, as follows: control group (n = 3), consisting of healthy uninfected children; group 1 (n = 4), comprising by HIV-1 paediatric patients who started cART very early (before 12 weeks of age); group 2 (n = 5), consisting of HIV-1 paediatric patients who started cART early (between 12 weeks and 1 year of age); and group 3 (n = 5), which included HIV-1 paediatric patients started cART late (after 1 year of age). The HIV-1 patients were receiving cART treatment (Tx), with different combinations of azidothymidine (AZT), lamivudine (3TC), nevirapine (NVP), abacavir (ABV), emtricitabine (FTC), efavirenz (EFV) and kaletra (KLT).

### Gene expression analysis

TaqMan low-density arrays (TLDA) results showed that 34 out of the 384 human miRNAs studied showed significant differences among the four groups examined (healthy control, very early HIV-1 patients were receiving cART treatment (Tx) initiation, early Tx initiation and late Tx initiation groups). Mentioned 384 human miRNAs commercially viable permitted coverage of Sanger miRBase v10 and represent the perfect solution for miRNA profiling applications. A summary of the F and p-values for one-way ANOVAs, as well as the subsequent results for the Tukey–Kramer post-hoc tests, are shown in Table 1.

**Table 1:** Summary of the results obtained from the statistical analysis performed on SPSS v.25. miRNAs presenting significant differences are shown. One Way-ANOVA F and p-values are shown, as well as those obtained from the Tukey–Kramer post-hoc tests between group means.

	One-way ANOVA		Post-hoc test (*p*-value)					
miRNA	F	General *p*-value	C-G1	C-G2	C-G3	G1–G2	G2–G3	G1–G3
*hsa-miR-27a*	4.54	0.024*	0.045*					0.032*
*hsa-miR-29a*	11.44	0.0008***	0.002**	0.003**			0.05*	0.033*
*hsa-miR-29c*	5.03	0.017*	0.014*	0.046*	0.055^			
*hsa-miR-34b*	7.57	0.004**			0.033*		0.004**	0.011*
*hsa-miR-107*	7.08	0.008**			0.032*	0.07^		0.011*
*hsa-miR-151-5p*	3.8	0.04*						0.046*
*hsa-miR-192*	8	0.004**				0.015*	0.003**	
*hsa-miR-202*	4.3	0.03*			0.027*			
*hsa-miR-301*	4	0.04*	0.026*					
*hsa-miR-320b*	4.1	0.033*	0.02*					
*hsa-miR-330*	5.02	0.018*	0.056^					0.038*
*hsa-miR-335*	4.12	0.032*						0.041*
*hsa-miR-346*	3.5	0.049*					0.032*	
*hsa-miR-365*	3.94	0.04*				0.032*		
*hsa-miR-509-5p*	3.97	0.035*			0.052^			0.064^
*hsa-miR-518e*	5.13	0.021*			0.06^		0.048*	0.033*
*hsa-miR-520d-3p*	4.65	0.022*				0.02*		0.07^
*hsa-miR-564*	3.97	0.038*	0.067^	* 0.05*				
*hsa-miR-571*	7.9	0.004**	0.06^				0.018*	0.004**
*hsa-miR-605*	5.85	0.012*					0.024*	0.012*
*hsa-miR-625*	5.96	0.01*					0.042*	0.006**
*hsa-miR-636*	5.16	0.016*					0.048*	0.011*
*hsa-miR-648*	5.94	0.014		0.018*			0.032	
*hsa-miR-650*	6.2	0.009**					0.006**	
*hsa-miR-1183*	4.57	0.024*					0.052^	0.025
*hsa-miR-1208*	4.76	0.021*						0.016
*hsa-miR-1225-3p*	8.85	0.0023**					0.004**	0.004**
*hsa-miR-1227*	9.9	0.0014**	0.04*				0.017*	0.0009***
*hsa-miR-1233*	7.7	0.004**					0.02*	0.0022**
*hsa-miR-1247*	7.9	0.004**			0.023*		0.052^	0.003**
*hsa-miR-1249*	3.93	0.04*					0.027*	
*hsa-miR-1254*	4.91	0.027*	0.051^			0.038*		
*hsa-miR-1275*	3.66	0.044*						0.049*
*hsa-miR-1303*	5.32	0.015*					0.042*	0.014*

C: Control; G1: Tx < 12 weeks; G2: Tx 12 weeks—1 year; G3: Tx > 1 year.

* *p* < 0.05; ** *p* < 0.01; *** *p* < 0.001; **^** tendency (*p* = 0.05–0.07).

**Figure 1:**
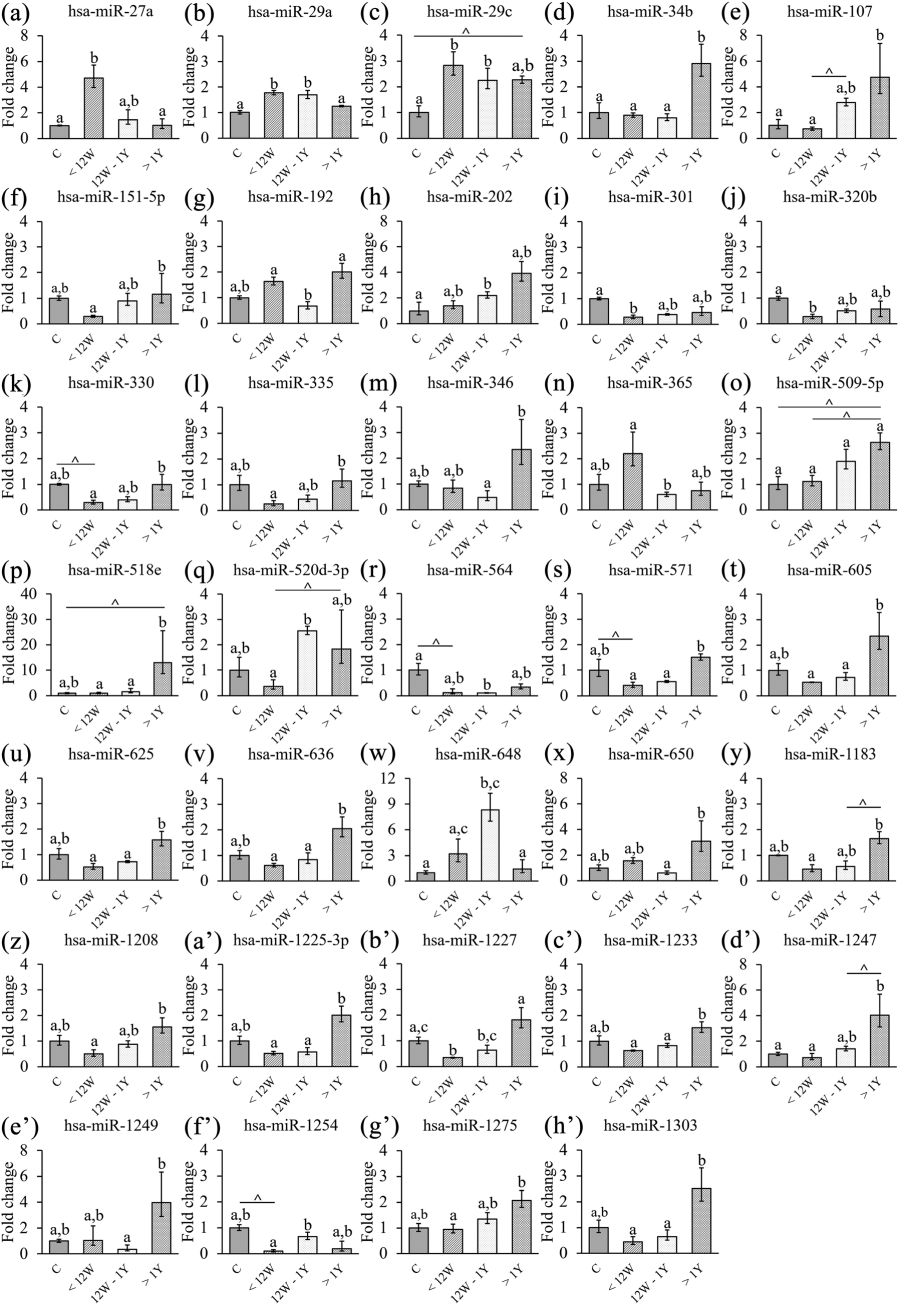
TaqMan low-density arrays (TLDA) arrays analysis of the expression levels of the 34 miRNAs showing statistical differences between groups (control (n = 3), Tx < 12 weeks (n = 4), Tx 12 weeks—1 year (n = 5) and Tx > 1 year (n = 4)). Data represent the fold-change value with respect to the control group mean value; mean ± SEM). Different lowercase letters indicate statistical differences exist between those groups (p < 0.05), while same letters indicate no statistical significance was found between those groups. * = p < 0.05 > 0.01; ** = p < 0.01 > 0.001; *** = p < 0.001. ^ means a tendency to statistical significance was found (p = 0.05–0.07). C = Control group; Tx < 12 weeks = Treatment started before 12 weeks of age; Tx 12 weeks—1 year = Treatment started between 12 weeks and 1 year of age; Tx > 1 year = Treatment stated after one year of age.

23 of these miRNAs exhibited a positive trend with later initiation of cART; only four of them presented a negative trend, and seven miRNAs showed an irregular trend, with the group starting cART between 12 weeks and 1 year either being over or under the expression levels of the rest of the treatment groups (Fig. 1).

In the group showing a positive trend, seven miRNAs started from the same or higher levels of expression as in the control group (miR-34b, miR-107, miR-202, miR-509-5p, miR-518e, miR-1247 and miR-1275), and 16 miRNAs presented an increasing trend starting below the expression levels of the control group (miR-151-5p, miR-301, miR-320b, miR-330, miR-335, miR-564, miR-571, miR-605, miR-625, miR-636, miR-1183, miR-1208, miR-1225-3p, miR-1227, miR-1233 and miR-1303) (Fig. 1).

The increasing trend in expression of these miRNAs suggests that cART is effective in controlling HIV-1 infection in these patients. For most miRNAs, the group starting cART at the earliest point showed the most similar levels of expression when compared to the control group, while the group starting cART at the point latest exhibited the least similar ones. These observations further suggest that HIV-1 infection of children activates expression of miRNA and the earlier the combination antiretroviral therapy can be started, the more effective it is in controlling the virus infection.

### Volcano plots

**Figure 2:**
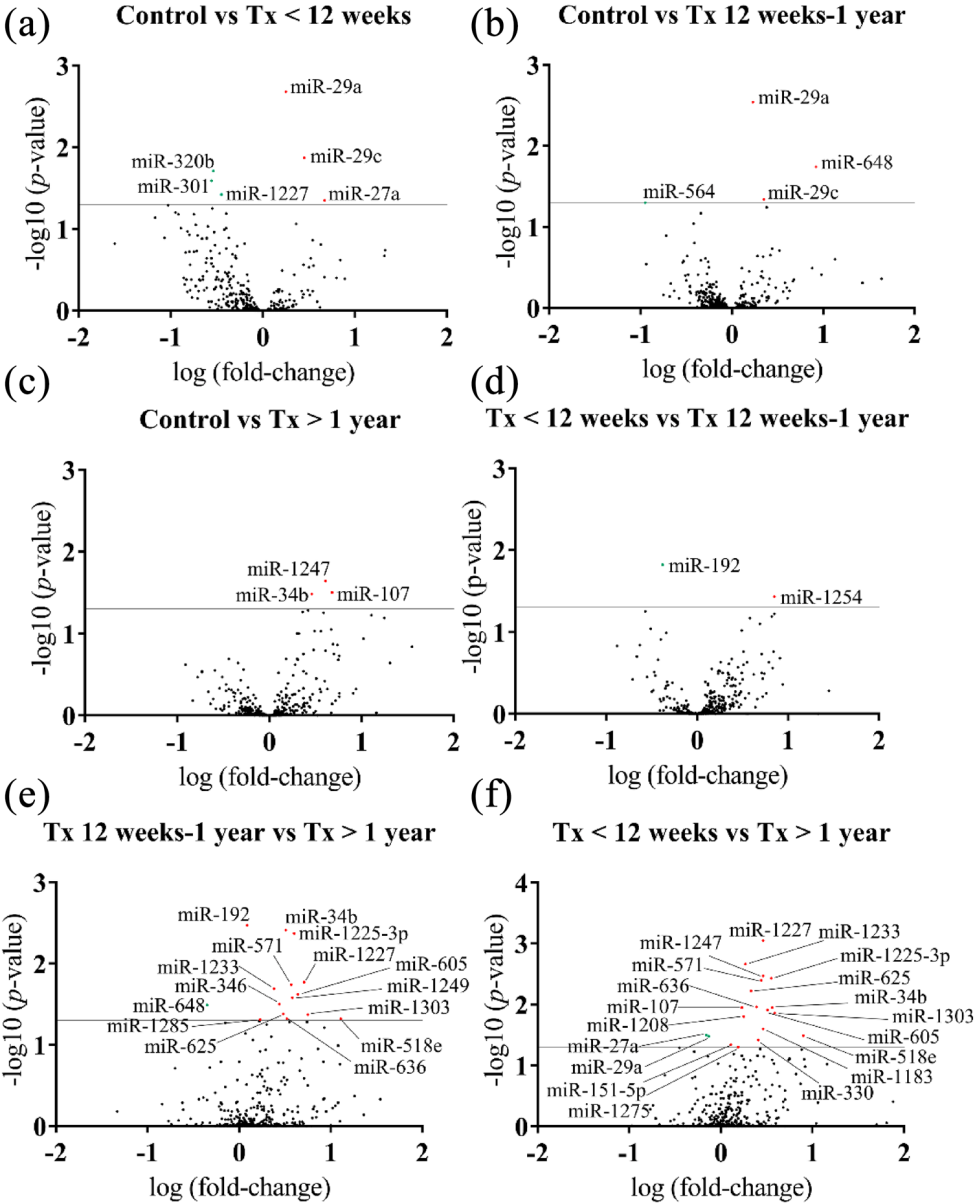
Volcano plots showing the fold change values for each of the 384 miRNAs studied organized between the various groups indicated on each plot. Downregulated genes are represented as green dots, while upregulated genes are depicted with red dots. Significant threshold was set at –log10 (p-value = 0.05). The most useful miRNAs can be found in the farthest points of the upper right and left corners.

Statistical significance obtained for these 34 miRNAs indicates useful but mostly mathematical probability that they will be helpful for diagnosis, prognosis or treatment follow-up for HIV-1 infection. Nonetheless, generating volcano plots provides a more complete interpretation of the data, as they provide information on potential biological usefulness. The larger the fold-change and p-value, the higher is the chance that the expression levels of the miRNAs will be biologically useful in the clinical field. Figure 2 shows the volcano plot graphs generated for each pair of groups which show the fold change value of each of the 384 dysregulated miRNAs in each group. In the case of the group starting cART before 12 weeks of age compared to the control group, miR-29a showed the greatest potential.

**Figure 3:**
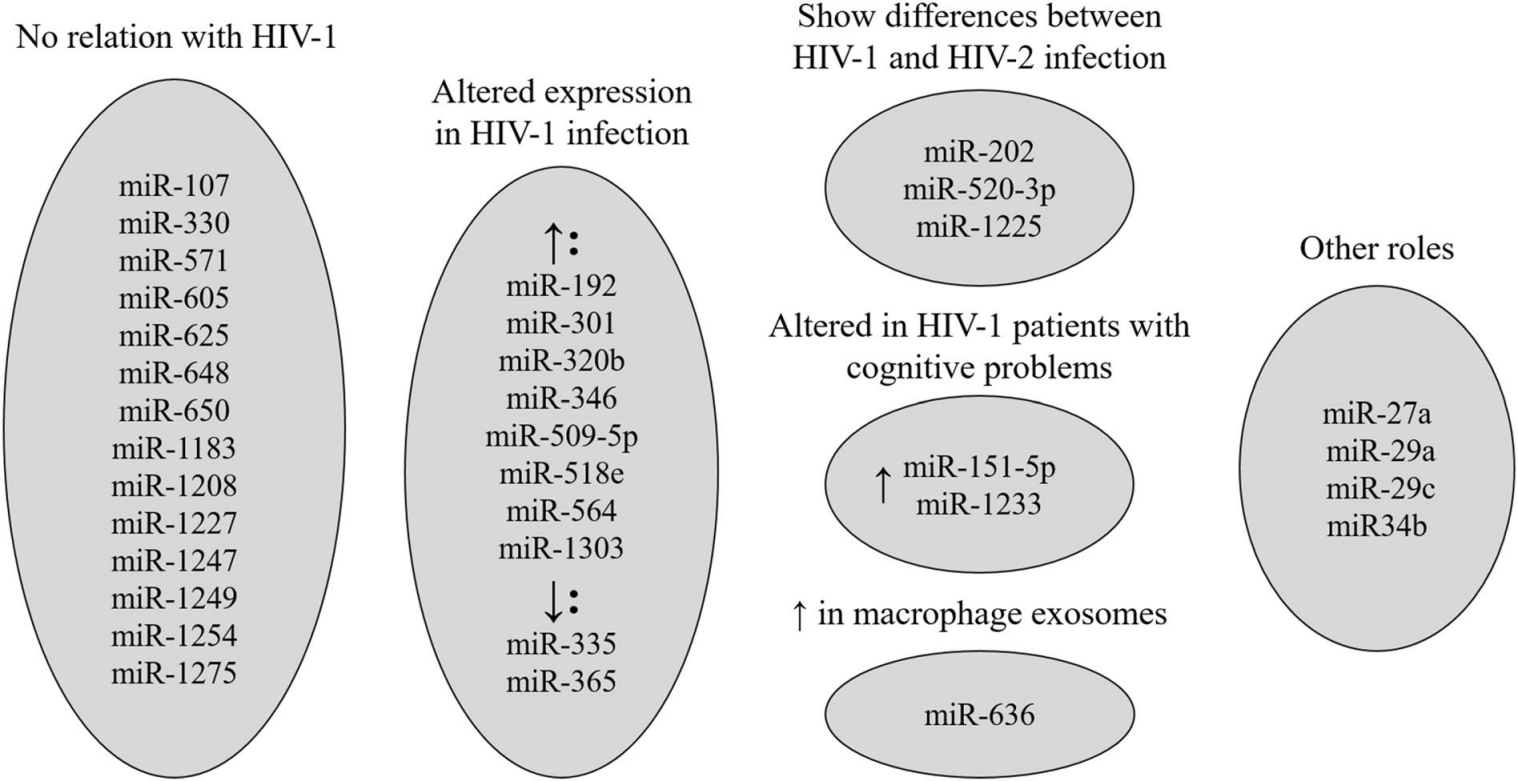
Graphical representation and categorization of the 34 miRNAs with statistically significant differences in relation to their general functions in HIV-1 infection.

The other miRNAs in this comparison were statistically significant but showed a lower fold-change value (Fig. 2a). With respect to the group starting cART between 12 weeks and 1 year of age in comparison to the control group, miR-29a again appeared as the most relevant one, followed by miR-648 (Fig. 2b). Finally, for the comparison between the group starting cART late, after 1 year of age, and the healthy control group, all four relevant miRNAs showed a small fold-change versus p-value relationship, with miR-1247 standing out as the potentially most useful miRNA (Fig. 2c). In relation to the three treatment groups, comparison between the group starting cART the earliest (before 12 weeks of age) and a little later (between 12 weeks and 1 year of age), revealed two miRNA, miR-192 and miR-520-3p, that showed downregulation and upregulation of their expression levels, respectively, in relation to their reference group (Fig. 2d). Finally, comparisons between the group starting cART early and that starting cART late (Fig. 2e), as well as between the group starting cART very early and the group late (Fig. 2f), showed the highest number of promising miRNAs and noncanonical volcano plot shapes, as derived from a higher variability of fold-change and p-values of the miRNAs in comparisons between these groups. miR-34b and miR-1225-3p appeared to be the most interesting miRNAs when comparing the early cART initiation group and the late cART initiation group, while miR-1227 and, again, miR-1225-3p were two of the most interesting miRNAs in the case of initiation of cART very early versus initiation very late. This is not to say that the other miRNAs do not have interesting data or may not be of interest in the clinical setting, but is rather to highlight those with the highest probability of having relevant biological relevance, possibly due to their functional roles, which are described in the text. In general, as clearly shown in Fig. 3, almost all the miRNAs studied among all groups showed an upregulation in their expression with respect to their reference groups.

When comparing the control and the very early treatment group, six miRNAs were over the 1.3 y axis threshold (equivalent to p = 0.05) (Fig. 2a). Half of them were upregulated and the other half downregulated in the treatment group compared to the control group. Four miRNAs were located over the threshold for early (Fig. 2b) and late (Fig. 2c) treatment compared to control group. The same trend was observed for the comparison between the early cART group and late cART group (Fig. 2d). The volcano plots for the comparisons between the early and late cART groups (Fig. 2) and between the very early and late cART groups (Fig. 2f) displayed the widest range of miRNA over the set threshold.

## Discussion

In this study, the data obtained from TLDA assays indicated the existence of a specific miRNA signature differentiating groups of pediatric patients starting cART at different times. As mentioned above, 34 of the 384 human miRNAs studied showed significant differences among the four groups (see Table 1 and Fig. 1). Our results indicate that, in general, pediatric patients who started cART later in life had a higher level of most of the 34 miRNAs identified and vice versa. A potential explanation for this observation is that a late HIV-1 infection diagnosis and establishment of an effective cART would allow HIV-1 to establish a large latent reservoir in the target cells, especially in CD4 + T cells^27,28^. Most research on alterations of host miRNAs due to HIV-1 infection has shown that expression levels of many of the miRNAs affected tend to be upregulated^8,29,30^. We found this to be true for the vast majority of the miRNAs we evaluated.

An increase in expression levels of miR-27a has been detected in plasma from HIV-1- infected adult patients compared to healthy controls^23^, possibly due to a decrease in phosphorylation of Akt and ERK, which negatively affects HIV-1 replication rate^31^. Surprisingly, in our case, the group starting cART the earliest showed a significant increase in miR-27a levels compared to the control group and the Tx groups starting cART later (Fig. 1a). It would be expected that lower miR-27a expression levels would result from earlier treatment after HIV infection, given that host cells would not need to increase expression of this miRNA to repress HIV-infection, as cART would be acting against it^32^. This effect, namely, an expression level comparable to that for healthy controls, was observed for both the group starting cART between 12 weeks and 1 year of age and after 1 year of age but not for the one starting treatment very early, as mentioned above. A possible explanation is that this increase in miR-27a expression may indicate a larger pool of resting CD4 + T cells in patients starting cART the earliest and that patients with delayed cART initiation would have more activated CD4 + T cells^32^. Another possibility relies on the fact that a previous work found that HIV-1 elite controller adult patients and healthy controls had higher expression of miR-27a, possibly indicating that very early initiation of cART has a positive effect and outcome in comparison to later cART initiation^33^.

miR-29 is one of the best studied miRNAs affecting HIV-1 infection course and therapy outcomes. Interestingly, we found significant differences in the expression levels of miR-29a, with the paediatric patients starting cART the earliest showing the highest expression levels and progressively lower levels for the paediatric group starting cART later (Fig. 1b). This negative association between progression of viraemia and delay of cART initiation has previously been reported in adult HIV-1-infected patients: researchers found that miR-29a correlated inversely with viral load and severity of immunosuppression^34^. A lower expression level of miR-29a also appears to suggest treatment failure in these HIV-1-infected patients^30,34^. In adult patients, miR-29a expression in PBMC was found to be downregulated by HIV-1 as viral the load increases and the disease progresses; in contrast, patients with upregulation of miR-29a showed a decreased viral load, probably due to the ability of this miRNA to hinder nef protein translation and to induce HIV-1 latency, hence reducing the HIV-1 load and infectivity^12,34^. In addition, miR-29a is very important in T-cells, being partly responsible for the activation of resting CD4 + T-cells^35^. The ability of miR-29a to bind HIV-1 nef gene transcripts is shared by other members of the miR-29 family, as they have the exact same seed sequence, even though their binding affinity and impact on HIV-1 infection is lower than that observed for miR-29a^36^. In this regard, we also found significant differences between patient groups for miR-29c, and the expression levels followed the same trend observed for miR-29a: the group starting cART before 12 weeks of age had significantly higher expression levels than the control group and the group starting cART after 1 year of age (Fig. 1b). Both miR-29a and miR-29c are reportedly downregulated in untreated HIV-1 patients^33,37^, thus matching to a great extent what we observed in paediatric patients untreated for longer than a year after HIV-1 infection establishment. Finally, miR-29a expression levels correlated with treatment success in adult patients, with those responding better to cART showing the highest levels^38^. Interestingly, miR-29a is useful as a prognostic biomarker in paediatric HIV-1 patients and for assessing treatment efficacy.

miR-34b also showed significant differences between the group means (Fig. 1c). miR-34a, another member of the miR-34 family, is upregulated in adult HIV-1-infected patients, as it promotes HIV-1 survival and replication^39,40^. It was suggested that this positive effect on HIV-1 pathogenesis is due to inhibition of the PNUTS/PPP1R10 protein complex by miR-34a, as the complex is detrimental for HIV-1 transcription by suppressing the interaction between cyclin T1 and CDK9, which is crucial for HIV-1 transcription^41^. In general, members of the same miRNA family will have similar functions, which would explain why miR-34b exhibited a positive trend in our samples, similar to that described for miR-34a by other authors in HIV-1 adult patients. Notably, while the groups of patients starting cART very early or early had miR-34b levels equivalent to those in the control group, patients starting cART later, over 1 year after diagnosis, showed elevated expression levels of this miRNA, suggesting a higher viral load and the potential establishment of a larger HIV-1 reservoir due to virus replication-beneficial miRNA-34b.

For the other miRNAs observed to be expressed significantly differently between the various groups of paediatric patients (30 out of 34, Fig. 1e–h’), we found little to no relationship between their function and HIV-1 infection in adult or paediatric HIV-1 patients. Specifically, 14 of those 30 miRNAs (miR-107, miR-330, miR-571, miR-605, miR-625, miR-648, miR-650, miR-1183, miR-1208, miR-1227, miR-1247, miR-1249 and miR-1254 and miR-1275) did not have any known relation with HIV-1 infection based on an extensive review of the scientific literature and different online databases. However, some of them have a role in other infectious diseases. For example, miR-1249 overexpression was shown to inhibit H5N1 infection^42^, and an increase in expression levels of miR-1275 is related to a high viral load of influenza A virus^43^. Interestingly, although no roles in HIV-1 infection were found for these fourteen miRNAs, 10 showed a positive trend among the groups we studied, beginning with the group in whom cART was initiated the earliest. Therefore, the later the paediatric patients started cART after the establishment of HIV-1 infection, the expression levels of these miRNAs progressively increased, even if viremia was undetectable. We hypothesize that these levels might constitute a response to the cART received or a result of larger active CD4 + and CD8 + T cell populations in the patients as cART initiation was delayed, given that the immune system in these patients would need to activate a larger pool of T cells to combat HIV-1 infection than in those starting cART very early^44^. The other four miRNAs with an unknown relationship with HIV-1 infection (miR-648, miR-650, miR-1249 and miR-1254) showed an irregular pattern among the three patient groups, with the group starting cART relatively early (between 12 weeks and 1 year) displaying higher expression levels than the other cART group. The reason for this irregular pattern is unknown, as no potential explanations were found in the scientific literature regarding the functional role of these miRNAs that could explain this irregular trend.

For the other 16 miRNAs, some information about a role in HIV-1 infection pathogenesis was found. Expression of eight of these miRNAs (miR-192, miR-301, miR-320b, miR-346, miR-509-5p, miR-518e, miR-564 and miR-1303) is increased in adult HIV-1 patients, usually as a result of viraemia^45–50^. Another two miRNAs (miR-335 and miR-365) were found to be decreased in HIV-1 patients^51^, with miR-335 showing lower levels in HIV-2-infected patients^52^. Three other miRNAs (miR-202, miR-520-3p and miR-1225) were reported to be useful for differentiating HIV-1 patients from those infected by HIV-2^52^ or detecting different levels of viremia in blood^24^. Two of the 16 miRNAs were altered in HIV-1 patients with cognitive impairment and helped to predict the appearance and progression of such cognitive problems^53,54^. Finally, miR-636 expression levels were increased in exosomes secreted by macrophages from it HIV-1-infected patients^55^. Figure 3 shows a summary of the roles of these 30 miRNAs.

Most of the miRNAs analysed and found to have some role or effect on HIV-1 infection displayed an increasing trend starting from an early initiation of cART to a late one. In addition, most of them were described in the literature as showing an increase in expression level either due to large viral loads, activation of T cells (especially CD4 + T cells) or an increase in the viral reservoir size^45–55^. These causes are usually tightly associated with respect to HIV replication in the host; therefore, the 34 dysregulated miRNAs appear to have potential prognostic usefulness as biomarkers by revealing not only the virological and immunological status at the beginning of treatment but also how the patient progresses while receiving cART. One exception to this increasing trend is miR-365, expression of which increased significantly in the early initiation cART group, but decreased in the groups starting cART later. Based on its generally decreased status in HIV-1 patients starting cART later in life in comparison to healthy controls, the reasons for its relative increase in pedriatric patients starting cART before 12 weeks after diagnosis are unexplainable to date, as pedriatric patients starting cART before 12 weeks after diagnosis also present a relative increase with respect to the healthy controls. In general, the other miRNAs followed the expected increasing trend based on the scientific literature for adult HIV-1 patients.

In summary, our results not only provide new data on miRNA status in paediatric HIV-1 patients, which had not been reported before, but also highlight the importance of starting cART as soon as possible after HIV-1 infection diagnosis, regardless of the age of the patient^44,56,57^ and follows the rationale that immediate cART initiation would avoid the creation of a large viral reservoir, which would be very difficult to tackle in the future; it may also help to avoid excessive T cell activation and subsequent depletion, an issue that would be highly challenging to reverse in adult life^58–60^. As expected, CD4 + T cell populations are more easily restored after effective cART, even if not initiated as soon as HIV-1 infection diagnosis is confirmed. However, CD8 + T cell counts are more difficult to recover if cART is not initiated as early as possible, thus further highlighting the urgent need to establish an effective therapy in paediatric patients, whose adaptive immune response is under development during that life period^60^. The 34 miRNAs found to have significant differences among groups starting cART at different time points after HIV-1 infection have great potential to be used as prognostic biomarkers. Nevertheless, given that the samples used in this study were from paediatric patients with undetectable viral load, a correlation between the expression levels of the 34 miRNAs and disease parameters, such as viral load, CD4 + and CD8 + T cell counts, severity of symptoms, and comorbidities, could not be established in the present work. Consequently, there is a window of opportunity to further investigate this miRNA panel in relation to a wide array of parameters to assess their real and quantitative usefulness as prognostic biomarkers in the follow-up of HIV-1 infection and immunological status. They could also serve as useful biomarkers to assess the effectiveness of ongoing or new therapies against HIV-1 infection.

In conclusion, we have established the first miRNA profile in PBMC of HIV-1 paediatric patients, differentiating them according to the time of cART initiation. It would have been better to have had patients in a narrower age range since the immune system of a 3-year old is very different from that of a 15-year old and this is a limiting factor of the study. As expected, the findings obtained from the data highlight the need to start cART as soon as possible after the establishment of HIV-1 infection and diagnosis confirmation, as the expression levels of most miRNAs indicated that a late cART establishment might be related to a larger HIV-1 reservoir size in latent CD4 + T cells, which may last during the lifetime of the patients, thus minimizing the possibilities of total virus eradication even in light of potential effective treatments in the near future. In addition, early cART initiation would avoid extensive activation of CD4 + and CD8 + T cells, which would make it very difficult to recover a normal T cell population and activation state, even with the application of effective cART. Thus, it is crucial to establish a correct cART as soon as the diagnosis confirms the HIV-1 infection. Finally, these miRNAs are attractive potential prognostic biomarkers useful for HIV-1 infection follow-up and assessment of therapy effectiveness, though more research must be performed to establish quantitative correlations.

## Methods

### Patient stratification

This study included 14 patients obtained from the HIV HGM BioBank (Madrid, Spain) (Biobank National Registry B.0000831 number), stablished in four groups: control group (n = 3), consisting of healthy uninfected children; group 1 (n = 4), comprising by HIV-1 paediatric patients who started cART very early (before 12 weeks of age); group 2 (n = 5), consisting of HIV-1 paediatric patients who started cART early (between 12 weeks and 1 year of age); and group 3 (n = 5), which included HIV-1 paediatric patients started cART late (after 1 year of age). Details of patients and treatments, ages and sex are defining in Table 2. Control group belong to a paediatric cohort of HIV HGM BioBank (Madrid, Spain) (Biobank National Registry B.0000831 number), with anonymized data.

**Table 2:** Patients stratification and group composition.

Patient code		Age (years)*	Sex	Treatment
Tx < 12 weeks	G1.1	3	F	AZT + 3TC + NVP
	G1.2	5	F	AZT + 3TC + NVP
	G1.3	7	F	AZT + 3TC + NVP
	G1.4	5	F	AZT + 3TC + NVP
Tx 12 weeks—1 year	G2.1	8	F	ABV + FTC + EFV
	G2.2	8	F	AZT + 3TC + NFV
	G2.3	10	M	ABV + FTC + KLT
	G2.4	8	F	AZT + 3TC + KLT
	G2.5	6	F	AZT + 3TC + NVP
Tx > 1 year	G3.1	7	M	AZT + 3TC + NFV
	G3.2	7	F	AZT + 3TC + NVP
	G3.3	5	M	AZT + 3TC + EFV
	G3.4	8	F	AZT + 3TC + NVP
	G3.5	15	M	AZT + 3TC + EFV

Tx < 12 weeks = Treatment started before 12 weeks of age; Tx 12 weeks – 1 year = Treatment started between 12 weeks and 1 year of age; Tx > 1 year = Treatment stated after one year of age. *Age at sample extraction time. F = Female; M = Male. AZT = azidothymidine, 3TC = lamivudine, NVP = nevirapine, ABV = abacavir, FTC = emtricitabine, EFV = efavirenz and KLT = kaletra.

All methods were carried out in accordance with relevant Spanish and EU guidelines and regulations as follows. PBMC samples were obtained from the HIV HGM BioBank (Madrid, Spain) (Biobank National Registry B.0000831 number), complying with the Ley 14/2007 and Real Decreto 1716/2011 regulations for the basic requirements of Biobank authorization and functioning for biomedical research purposes with human samples.

Clinical data from the patients were stored in the National Cohort of HIV-1 infected children (CoRISpe) node network, assuring the patients’ confidentiality rights by complying with the Ley Organica 15/1999 for the protection of personal information. The donors, as well as their parents and/or guardians, signed an informed consent form for the use of the samples and data for research purposes, according Ley 14/2007 guideline for the regulation of biomedical research.

Experimental protocols with human samples were approved by the Ethics and Experimental Committee of Hospital Universitario Gregorio Marañón with project code miRNA-Ped-HIV_16-01.

### Sample collection, processing and RNA isolation

Blood samples were collected in sterile EDTA tubes. Plasma was separated and stored, as were PBMCs, by using the Ficoll-Hypaque (FP) gradient centrifugation method. Upon initiation of the experiments, PBMCs were activated with phytohemaglutinin (PHA) (Sigma-Aldrich, St. Louis, MO, USA) for 72 h, washed and pelleted. Isolation of RNA was performed with mirVana miRNA isolation kit (Ambion, Huntingdon, UK), following the manufacturer’s instructions. RNA concentrations were measured with a Nanodrop ND-1000 (Thermo Scientific, Waltham, MA, USA) and RNA quality and integrity (RIN values) were assessed with an Agilent 2100 Bioanalyzer (Agilent Technologies, Santa Clara, CA, USA).

### miRNA profiling with TaqMan low-density arrays (TLDA)

RNA resulting in previous isolation (1–350 ng) were reverse-transcribed with the MicroRNA Reverse Transcription Kit (Ambion, Huntingdon, UK), in combination with the stemloop Megaplex RT Primers (Ambion, Huntingdon, UK), which allows reverse transcription of many small RNAs at the same time. The miRNA gene expression profile was assessed using TaqMan Array Human microRNA Card A v2.0 (Applied Biosystems, Foster City, CA, USA) containing 384 human miRNAs (hsa-miR) and the Applied Biosystems 7900HT Fast Real-Time PCR system.

### Statistical analysis

Data obtained from the TLDA assay were analyzed following the ΔΔCt method developed by Livak and Schmittgen^25^. Samples with amplification cycles over 38 were removed and considered not amplified. The geometric mean of RNU44, RNU48 and U6 expression levels was used as an endogenous control to normalize the results of the target genes. Data are expressed as the fold change with respect to the control group. Statistical differences between group means were analyzed with one-way ANOVA after checking the assumptions of normality, homogeneity of variances and independence of the samples using SPSS 25 Statistics software (IBM, NY, USA). Comparisons between all four groups were performed (16 combinations in total). Statistical significance was established for p-values below 0.05 (* p < 0.05). Further statistically significant p-values were established as ** p < 0.01 and *** p < 0.001. miRNAs with significant ANOVA results were further assessed with the Tukey–Kramer post-hoc test to detect and analyse specific differences between each group mean. Data are expressed as the fold-change ± S.E.M.

### Volcano plots

Volcano plot graphs were produced for a better analysis of the most interesting miRNAs from a biological standpoint. The x axis represents the log(fold-change) and the y axis the − log10(p-value) of the data for each comparative group. The dark gray line represents the − log10(p-value = 0.05) threshold, which was set at 1.3. miRNAs with values located in the upper right showed upregulation (red dots) compared with the reference group; those located in the upper left corner (green dots) showed downregulation compared with the reference group. miRNAs were considered to be more biologically relevant the farther they were located from the intercross between the x axis origin and y threshold. The volcano plots were generated using GraphPad Prism v.5 (San Diego, California, USA).
